# Significant Incidental Findings in the National Lung Screening Trial and Diagnosis of Extrapulmonary Cancer

**DOI:** 10.1001/jamanetworkopen.2026.3398

**Published:** 2026-03-31

**Authors:** Ilana F. Gareen, Roee Gutman, Maryanne Thangarajah, Amal N. Trivedi, Tina D. Tailor, Efren Flores, Caroline Chiles, JoRean Sicks, Richard M. Hoffman

**Affiliations:** 1 Department of Epidemiology, Brown University School of Public Health, Providence, Rhode Island; 2 Center for Biostatistics and Health Data Science, Brown University School of Public Health, Providence, Rhode Island; 3 Department of Biostatistics, Brown University of Public Health, Providence, Rhode Island; 4 Department of Health Services, Policy and Practice, Brown University School of Public Health, Providence, Rhode Island; 5 Center of Innovation for Long-Term Services and Supports, Providence VA Medical Center, Providence, Rhode Island; 6 Division of Cardiothoracic Radiology, Department of Radiology, Duke Health, Durham, North Carolina; 7 Department of Radiology, Massachusetts General Hospital, Boston; 8 Department of Radiology, Atrium Health Wake Forest Baptist, Winston-Salem, North Carolina; 9 Department of Internal Medicine, University of Iowa Carver College of Medicine, Iowa City; 10 Holden Comprehensive Cancer Center, University of Iowa Health Care, Iowa City

## Abstract

**Importance:**

Significant incidental findings (SIFs) not related to lung cancer have been widely reported in patients undergoing lung cancer screening with low-dose computed tomography (LDCT). It is unclear whether SIFs are associated with extrapulmonary cancer diagnoses.

**Objective:**

To examine the association between an SIF considered to be potentially indicative of extrapulmonary cancer (cancer SIF) detected at LDCT lung cancer screening and diagnosis of an extrapulmonary cancer within 1 year of the screen.

**Design, Setting, and Participants:**

This retrospective cohort study analyzed data from National Lung Screening Trial (NLST) participants. The NLST participants were randomly assigned to either LDCT or chest radiography to determine whether LDCT was associated with a reduction in lung cancer mortality compared with chest radiography alone. Participants aged 55 to 74 years were recruited between August 2002 and April 2004. They received up to 3 rounds of screening and were followed up for 5 to 7 years. The study concluded December 31, 2009. This analysis was restricted to participants in the LDCT arm and was conducted between June and December 2025.

**Exposure:**

Detection of a cancer SIF (ie, SIF potentially indicative of a cancer) at any lung cancer screening round in the NLST.

**Main Outcomes and Measures:**

The primary outcome was diagnosis of an extrapulmonary cancer within 1 year of a screening round. Extrapulmonary cancers were classified using Surveillance, Epidemiology, and End Results (SEER) Program organ system categories. Cancer SIFs were mapped to specific SEER cancer categories. Multilevel logistic regression was used to assess the association between detection of a cancer SIF and diagnosis of an extrapulmonary cancer.

**Results:**

The study included 75 104 LDCT screening rounds performed in 26 445 participants (mean [SD] age, 61.4 [5.0] years; 59.0% male). Cancer SIFs were reported for 2265 screening rounds (3.0%) in 1807 participants (6.8%) across the 3 screening rounds. An extrapulmonary cancer was diagnosed following a screening round with a cancer SIF (n = 2265) for 67 participants (3.0%). The marginal risk difference, after covariates and participant-specific adjustments, was 13.89 (95% confidence limit [CL], 7.03-20.75) per 1000 participants. The marginal risk differences were significantly higher for urinary cancers (17.03 [95% CL, 8.55-25.50] per 1000 participants) and other SEER cancer categories, including lymphoma and leukemia (13.83 [95% CL, 3.46-24.21] per 1000 participants).

**Conclusions and Relevance:**

This cohort study found that cancer SIFs were associated with an increased risk of an extrapulmonary cancer diagnosis in the year following an LDCT lung cancer screening examination. These findings suggest that certain SIFs should be evaluated as potential indicators of undiagnosed cancers.

## Key Points

**Question:**

What was the association between significant incidental findings (SIFs) detected on low-dose computed tomography screening examinations in the National Lung Screening Trial and extrapulmonary cancer diagnoses?

**Findings:**

In this cohort study of 26 445 participants, those in whom a cancer-associated SIF was detected had a higher risk of cancer diagnosis in the year following the screening examination than participants with no cancer-associated SIF.

**Meaning:**

These findings suggest that certain SIFs should be evaluated as potential indicators of undiagnosed cancers.

## Teaser

This cohort study examines the association between significant incidental findings on lung cancer screening in the National Lung Screening Trial and the diagnosis of an extrapulmonary cancer within 1 year of the screen.

## Introduction

Significant incidental findings (SIFs) are an important issue in medical imaging. Low-dose computed tomography (LDCT) lung cancer screening frequently detects SIFs unrelated to lung cancer.^1,2,3^ These findings have potential clinical significance and are of sufficient concern that the radiologist informs the referring physician. In the National Lung Screening Trial (NLST), 8954 of 26 455 participants (33.8%) screened with LDCT had SIFs reported. The nature of the SIFs varied, though more than 10% of SIFs were potentially indicative of an extrapulmonary cancer.^4^

While current recommendations^5,6,7,8,9,10,11,12,13^ exist for reporting and addressing SIFs, the evidence base examining the association between SIFs detected at LDCT lung cancer screening with an extrapulmonary cancer diagnosis is limited. The population undergoing lung screening comprises patients with a history of heavy smoking who are at high risk of several extrapulmonary cancers, including pancreatic, bladder, and kidney cancer.^14,15,16^ Previous publications have reported that abnormalities below and above the diaphragm and SIFs in general are associated with kidney cancer^17,18^ and thyroid cancer^18,19^ diagnoses. The power of some of these analyses may have been limited, as they focused on all SIFs as opposed to those potentially related to cancer.^18,19^ The improved ability to detect an association between SIFs indicative of cancer and a related cancer diagnosis was apparent in a report by Pinsky et al^17^ that kidney cancer was more common in participants with kidney masses as opposed to those without. Furthermore, while Pinsky et al^17^ adjusted for age, sex, smoking status, and pack-years smoked, other authors did not adjust for potential confounders.^18,19^

Gareen et al^4^ recently classified text comments from NLST case report forms describing SIFs detected at LDCT lung cancer screening, using criteria described in American College of Radiology white papers.^6,7,8,9,10,11,12,13^ These SIF classifications provide an opportunity to examine the potential association between the detection of SIFs that may be indicative of a cancer (hereafter referred to as cancer SIFs) and diagnosis of extrapulmonary cancer. We linked NLST data, including information on baseline history of comorbidities and extrapulmonary cancers, with the Surveillance, Epidemiology, and End Results (SEER) Program organ system cancer categories^20^ to examine whether the rate of cancer in the year following screening was higher for patients with cancer SIFs than in those without cancer SIFs.

## Methods

This cohort study used data collected for the NLST, a collaboration between the American College of Radiology Imaging Network, now part of the Eastern Cooperative Oncology Group–American College of Radiology Imaging Network Cancer Research Group, and the Lung Screening Study. We made a formal data request to the Cancer Data Access System for data.^21^ This study was reviewed by the Brown University Institutional Review Board and received an expedited review and approval under expedited category 5. The study followed the Strengthening the Reporting of Observational Studies in Epidemiology (STROBE) reporting guideline for cohort studies.

The NSLT has been described in detail elsewhere.^22,23,24^ In brief, the NLST was a multi-institutional trial of 53 452 participants recruited between August 2002 and April 2004 and concluded December 31, 2009. The trial was designed to determine whether screening with LDCT was associated with a reduction in lung cancer mortality compared with chest radiography. Patients eligible for the NLST were aged 55 to 74 years at study accrual, had a cigarette smoking history of at least 30 pack-years, and were either currently smoking or quit smoking within the previous 15 years. Patients were excluded if they had a history of lung cancer, hemoptysis, or unexplained weight loss in the preceding year or if they had been treated for cancer or been told by a physician that they had evidence of cancer (excluding nonmelanoma skin cancer or in situ cancers other than in situ transition cell or bladder cancers) in the past 5 years.^22^ Participants were randomly assigned to LDCT or chest radiography and were scheduled to receive 3 rounds of screening at 1-year intervals. Participants were followed up for 5 to 7 years from recruitment, and detailed information on any cancer diagnosis and cause of death was collected.^22,23,24^ Inclusion and exclusion criteria are provided in eTable 1 in Supplement 1. For this report, we focused on the participants randomized to the LDCT arm of the trial.

### Patient Population

We included all NLST participants randomized to the LDCT arm of the trial who had at least 1 screening round. Using information from NLST case report forms, we examined screening results from each individual screening test. We also examined whether participants ever had a cancer SIF reported across the 3-round screening program. Participants diagnosed with lung cancer prior to the first screening round were excluded from our analyses.

### Exposure of Interest

The process used to interpret NLST LDCT screening rounds, describe SIFs, and assign a final diagnosis was described previously.^4^ In brief, the final result at each screening visit was classified as either a positive (suspicious for lung cancer) or negative (no findings suggestive of lung cancer) screen. Participants with a negative screen were further classified as having a negative screen with significant abnormalities not suspicious for lung cancer (SIFs) or a negative screen with no significant abnormalities. Participants with a positive screen may also have had SIFs detected.

For participants with a positive screen, or with a negative screen with significant abnormalities not suspicious for lung cancer, the type of SIF was characterized on case report forms as (1) emphysema, (2) a significant cardiovascular abnormality, (3) an other potentially significant abnormality above the diaphragm, or (4) an other potentially significant abnormality below the diaphragm. Radiologists had the option of specifying the nature of the abnormality for cardiovascular abnormalities and were asked to specify the nature of the abnormality for those above or below the diaphragm. These descriptions of abnormalities were entered as free text. In an earlier study,^4^ these free-text SIFs were classified using American College of Radiology white paper criteria.^4,5,6,7,8,9,10,11,12,13^

Using the categorized SIFs that were considered reportable to the referring clinician,^4^ our medical panel of 3 physicians (A.N.T., T.D.T., and R.M.H.) reached consensus on those SIFs that were potentially related to a cancer diagnosis (cancer SIF), ie, findings that they would want to have reported to them for further investigation. A.N.T. and R.M.H. specialize in internal medicine, and T.N.T. is a radiologist. The exposure of interest for this analysis was detection of a cancer SIF at lung cancer screening. The specific cancer SIFs are shown in eTable 2 in Supplement 1.

After screening, results were sent to the community practitioner on record, and participants followed up with their physician. For extrapulmonary cancers, information on diagnostic follow-up and treatment was not routinely collected as part of the NLST.

### Outcomes and Measures

Information on extrapulmonary cancers was collected in the NLST as part of the standard follow-up procedure. Medical records for all cancers were requested by site research associates and provided to certified tumor registrars who assigned *International Classification of Diseases for Oncology, 3rd Edition* codes. These codes were then categorized using the SEER cancer classifications in the Cancer Data Access System database.^21,25^ We evaluated the association between SIFs and SEER cancer categories, rather than specific cancers, to increase the number of cancers in each analysis and allow us to adjust for important covariates.

The primary outcome for this study was diagnosis with an extrapulmonary cancer in the year following a screening round. Screening rounds were scheduled to occur at 1-year intervals, and we operationalized this as diagnosis before the next screening round or, in the absence of another screen, at 1 year from the index screening round. For simplicity, the term *within 1 year of the screening round *is used to define the period of interest.

### Linkage of Cancer SIFs With SEER Categories

Each cancer SIF was matched with the SEER cancer classification for which the SIF was considered indicative of a potential neoplasm. Examples of cancer SIFs included kidney masses, pancreatic masses, and breast nodules. Certain cancer SIFs, such as enlarged lymph nodes, could be nonspecific. The potential impact of these nonspecific SIFs was considered in the primary analysis evaluating the association between a cancer SIF and any extrapulmonary cancer and in the assessment for an association with other SEER cancer categories not otherwise evaluated. The cancer SIF–SEER classification linkages are shown in eTable 2 in Supplement 1.

### Covariates

The NLST collected information on demographic, medical, and health history at accrual into the trial.^22^ Covariates included sex, age, race (self-reported Black, White, or other [American Indian or Alaska Native, Asian, Native Hawaiian or Pacific Islander, multiracial, or unknown; category collapsed due to data being too sparse to obtain robust estimators of the effect for these groups]), ethnicity (self-reported Hispanic or Latino, non-Hispanic or Latino, or unknown), education, marital status, smoking status, smoking pack-years, occupational history, smoking-related medical history, and history of extrapulmonary cancer diagnosed during the 5 years before study entry. Details on the occupational, medical, and cancer history variables collected are provided in eTable 3 in Supplement 1. Because of the large number of potential work covariates and the small number of cancers, we combined all occupational history into a history of high-risk work (yes, no) to achieve identifiability. Similarly, we adjusted for history of an extrapulmonary cancer (yes, no) and history of medical conditions (yes, no).

### Statistical Analysis

The data analysis was performed between June and December 2025. We compared demographic characteristics for participants with a cancer SIF detected at any screen during the trial with those who never had an SIF detected using the *t* test for continuous variables and χ^2^ test for categorical variables.

To assess the association between cancer SIF detection and extrapulmonary cancer diagnosis, and to capture any potential effect of nonspecific cancer SIFs, we first examined whether there was an association between any cancer SIF and any extrapulmonary cancer. We adjusted for the aforementioned covariates. We then examined the association between the specific cancer SIFs and the specific SEER cancer categories hypothesized to be related to them as detailed in eTable 2 in Supplement 1. The SEER cancer categories for which the sample size was small (eg, endocrine cancers) were grouped into an other cancer category. We examined the association between these other cancers and all cancer SIFs not related to another SEER cancer category.

We developed a multilevel logistic regression model to assess the association between detection of a cancer SIF and an extrapulmonary cancer. In the first level of this model, we adjusted for the aforementioned covariates, including a history of cancer prior to enrollment in the NLST, as well as screening time point and an interaction between cancer SIF and screening time point. In the second level, we adjusted for a participant-specific intercept to account for the repeated screening of the same participant.

Using the model, we estimated the marginal difference in risks of any cancer SIF and any extrapulmonary cancer and for specific SEER cancer groups with and without the detection of a cancer SIF related to that SEER cancer group. Because of the smaller number of cancers in the regressions evaluating the risk of specific SEER cancer categories hypothesized to be related to specific cancer SIFs (eTable 2 in Supplement 1), we were unable to adjust for the interaction between cancer SIF and screening time point. In addition, in the analyses examining urinary, digestive, and breast cancer, we were unable to adjust for ethnicity or marital status because of identifiability issues. For breast cancer, sex was also excluded from the model. We used SAS, version 9.4 (SAS Institute Inc) and R, versions 4.5.0, 4.5.1, and 4.5.2 (R Foundation for Statistical Computing). The threshold for significance was a 2-sided *P* < .05.

## Results

The study population included 26 445 participants (mean [SD] age, 61.4 [5.0] years; 41.0% female and 59.0% male; 4.5% identifying as Black, 91.2% as White, and 4.4% as other race; 1.8% identifying as Hispanic or Latino, 97.8% as non-Hispanic or Latino, and 0.4% as unknown ethnicity) with at least 1 LDCT screening round (Table 1; eFigure in Supplement 1). The 75 104 LDCT lung cancer screening rounds detected 2558 cancer SIFs at 2265 cancer screening rounds in 1807 participants. A total of 235 participants (13.0% of 1807 with a cancer SIF) had more than 1 cancer SIF at the same screening round, 331 (18.3% of 1807 with a cancer SIF) had the same cancer SIF reported at multiple screening rounds, and 103 (5.7% of 1807 with a cancer SIF) had a different type of cancer SIF detected at different screening rounds. At least 3.0% of screening rounds (2265 of 75 104) had at least 1 cancer SIF detected; and 6.8% of participants (1807 of 26 445) had at least 1 cancer SIF detected over the course of up to 3 screening rounds.

**Table 1.** Demographics Characteristics of NLST LDCT Arm Participants With a Cancer-Associated SIF Reported at Least Once During Screening vs Those Who Did Not

Characteristic	Participants, No. (%)^a^		*P* value
	Cancer SIF (n = 1807)	No cancer SIF (n = 24 638)	
Age, mean (SD), y	62.1 (5.1)	61.4 (5.0)	<.001
Smoking pack-years, mean (SD)	55.6 (23.4)	56.0 (24.0)	.41
Race			
Black	90 (5.0)	1087 (4.4)	.25
White	1649 (91.3)	22 467 (91.2)	
Other^b^	68 (3.8)	1084 (4.4)	
Ethnicity			
Hispanic or Latino	22 (1.2)	448 (1.8)	.14
Non-Hispanic or Latino	1776 (98.3)	24 092 (97.8)	
Unknown or refused to answer	9 (0.5)	98 (0.4)	
Sex			
Female	749 (41.4)	10 083 (40.9)	.66
Male	1058 (58.6)	14 555 (59.1)	
Education			
≤11th Grade	112 (6.2)	1513 (6.1)	.04
High school graduate or GED	435 (24.1)	5778 (23.5)	
Post–high school training, excluding college	261 (14.4)	3444 (14.0)	
Associate’s degree or some college	429 (23.7)	5711 (23.2)	
Bachelor’s degree	263 (14.6)	4202 (17.1)	
Graduate school	256 (14.2)	3506 (14.2)	
Other^c^	51 (2.8)	484 (2.0)	
Marital status at the start of trial			
Never married	91 (5.0)	1152 (4.7)	.34
Married or living as married	1218 (67.4)	16 472 (66.9)	
Widowed	148 (8.2)	1821 (7.4)	
Separated	16 (0.9)	318 (1.3)	
Divorced	329 (18.2)	4803 (19.5)	
Other^d^	5 (0.3)	72 (0.3)	
Smoking status			
Former	917 (50.7)	12 810 (52.0)	.31
Current	890 (49.3)	11 828 (48.0)	
History of work in any high-risk occupation			
Yes	484 (26.8)	6898 (28.0)	.27
No	1323 (73.2)	17 740 (72.0)	
History of potentially smoking-related disease			
Yes	1239 (68.6)	16 180 (65.7)	.01
No	568 (31.4)	8458 (34.3)	
History of any extrapulmonary cancer			
Yes	84 (4.6)	988 (4.0)	.18
No	1723 (95.4)	23 650 (96.0)	

Abbreviations: GED, General Educational Development; LDCT, low-dose computed tomography; NLST, National Lung Screening Trial; SIF, significant incidental finding.

^a^ Due to rounding, percentages may not add to 100.

^b^ Included American Indian or Alaska Native, Asian, Native Hawaiian or Pacific Islander, multiracial, unknown, or refused to answer.

^c^ Included other education or unknown.

^d^ Included refused to answer, not ascertained, or missing.

Table 1 shows demographic characteristics of LDCT participants by cancer SIF status. Participants were classified as ever having a cancer SIF detected on at least 1 screening round (n = 1807) or not (n = 24 638). Participants with cancer SIFs were significantly older compared with those with no cancer SIF (mean [SD], 62.1 [5.1] vs 61.4 [5.0] years; *P* < .001) and more likely to have a history of a smoking-related disease (68.6% vs 65.7%; *P* = .01). Detailed information on occupational and medical history is shown in eTable 3 in Supplement 1.

Table 2 shows the risk of an extrapulmonary cancer diagnosis within 1 year of a cancer SIF. Overall, 1025 participants were diagnosed with at least 1 extrapulmonary cancer within 1 year of a screening round; 11 of these participants (1.1%) had 2 extrapulmonary cancers diagnosed following 2 separate screening rounds (1036 total cancer diagnoses). Twelve participants had 2 cancers diagnosed following a single screening visit. Sixty-seven of 1025 participants (6.5%) diagnosed with an extrapulmonary cancer within 1 year of a screening round had a cancer SIF. The absolute risk difference of being diagnosed with an extrapulmonary cancer within 1 year of a cancer SIF compared with no cancer SIF was 16.28 per 1000 participants. The marginal risk difference per 1000 participants was 13.89 (95% confidence limit [CL], 7.03-20.75) higher among those with a cancer SIF compared with no cancer SIF. eTable 4 in Supplement 1 shows the full model with all covariates.

**Table 2.** Number of Screening Rounds With at Least 1 Cancer SIF Detected and Participants With at Least 1 Cancer Diagnosed Within 1 Year of a Screening Round (n = 1025)

Cancer SIF	No. of screening rounds with ≥1 cancer SIFs detected (n = 75 104)	No. of cancers diagnosed within 1 year of a screening round (n = 1036)^a^	Risk of extrapulmonary cancer, per 1000 screening rounds	Crude risk difference, per 1000 participants^b^	Marginal risk difference (95% CL), per 1000 participants^c^
Yes	2265	67	29.58	16.28	13.89 (7.03-20.75)
No	72 839	969	13.30	NA	NA

Abbreviations: CL, confidence limit; NA, not applicable; SIF, significant incidental finding.

^a^ There were 11 participants diagnosed with cancer at 2 different time points.

^b^ Unadjusted.

^c^ Adjusted for sex, age, race, ethnicity, education, marital status, smoking status, smoking pack-years, occupational history, smoking-related medical history, history of any cancer diagnosed during the 5 years prior to study entry, screening time point, interaction between cancer SIF and screening time point, and repeated screening for the same participant.

The Figure shows the unadjusted absolute risk of SEER cancer categories by related cancer SIF status. Table 3 shows the association between cancer SIFs and the related SEER cancer categories. There were 148 urinary cancers, 169 digestive cancers, 152 breast cancers, and 579 other cancers. As previously described, some participants had more than 1 cancer SIF at a single screening test. The marginal risk difference per 1000 participants of being diagnosed with an extrapulmonary cancer within 1 year of having a related cancer SIF was 17.03 (95% CI, 8.55-25.50) for urinary cancer, 5.02 (95% CL, −1.33 to 11.36) for digestive cancer, 12.30 (95% CL, −3.87 to 28.48) for breast cancer, and 13.83 (95% CL, 3.46-24.21) for other cancers. The full models are shown in eTables 5 to 8 in Supplement 1. Only cancers related to SIFs as described in eTable 2 in Supplement 1 were included in these analyses. Excluded from these analyses were 24 cancers diagnosed in patients with unrelated SIFs.

**Figure.**
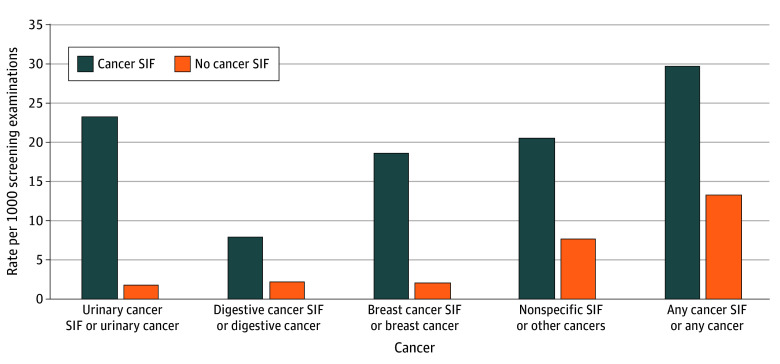
Bar Graph Showing the Unadjusted Rate Per 1000 Screening Rounds of Surveillance, Epidemiology, and End Results (SEER) Cancer Group Diagnoses by Associated Cancer Significant Incidental Finding (SIF) The other cancer category includes SEER cancer categories for which the sample size was small (eg, endocrine cancers).

**Table 3.** Number of Screening Rounds With at Least 1 Cancer SIF Related to a SEER Cancer Category and Extrapulmonary Cancers Diagnosed Within 1 Year of a Screening Round^a^

SIF and related SEER cancer category	No. of screening rounds with ≥1 matched cancer SIF detected	No. of cancers diagnosed within 1 year of a screening round^b^	Risk of related extrapulmonary cancer per 1000 screening rounds	Crude risk difference, per 1000 participants^c^	Marginal risk difference (95% CL), per 1000 participants
Urinary cancer					
Yes	819	19	23.20	21.46	17.03 (8.55 to 25.50)^d^
No	74 285	129	1.74	NA	NA
Digestive cancer					
Yes	646	5	7.74	5.53	5.02 (−1.33 to 11.36)^d^
No	74 458	164	2.20	NA	NA
Breast cancer					
Yes	161	3	18.63	16.65	12.30 (−3.87 to 28.48)^e^
No	74 943	149	1.99	NA	NA
Other cancer					
Yes	781	16	20.49	12.91	13.48 (3.46 to 24.21)^f^
No	74 323	563	7.58	NA	NA

Abbreviations: CL, confidence limit; NLST, National Lung Screening Trial; NA, not applicable; SEER, Surveillance, Epidemiology, and End Results; SIF, significant incidental finding.

^a^ The NLST participants may have had up to 3 screening rounds. Included are 75 104 screening rounds. More than 1 cancer SIF might have been detected at a screening round, in which case, the participant may appear in more than 1 SEER cancer category. Twenty-three participants were diagnosed with more than 1 extrapulmonary cancer.

^b^ Does not include 24 extrapulmonary cancers without a directly related SIF per eTable 1 in Supplement 1.

^c^ Unadjusted.

^d^ Adjusted for sex, age, race, education, smoking status, smoking pack-years, occupational history, smoking-related medical history, history of any cancer diagnosed during the 5 years prior to study entry, screening time point, and repeated screening for the same participant.

^e^ Adjusted for age, race, education, smoking status, smoking pack-years, occupational history, smoking-related medical history, history of any cancer diagnosed during the 5 years prior to study entry, screening time point, and repeated screening for the same participant.

^f^ Adjusted for sex, age, race, ethnicity, education, smoking status, smoking pack-years, marital status, occupational history, smoking-related medical history, history of any cancer diagnosed during the 5 years prior to study entry, screening time point, and repeated screening for the same participant.

Pretrial cancer history was not significantly associated with extrapulmonary cancer diagnoses (eTables 4-8 in Supplement 1). Of patients with a cancer SIF who were diagnosed with a related cancer during the trial, a history of that same cancer was reported at baseline for 2 of 19 urinary cancers (10.5%), 1 of 3 breast cancers (33.3%), and 0 digestive or other cancers.

Detailed information on the type of cancer SIF and the occurrence of these SIFs by screening round and per participants across 3 screening rounds, as well as the number and type of cancers related to each SIF, are shown in eTable 9 in Supplement 1. Detailed information on the type of cancer SIF, diagnosis of an extrapulmonary cancer within 1 year of the screening round, and deaths due to extrapulmonary cancer during the course of the trial is presented in eTable 10 in Supplement 1. The total number of extrapulmonary cancers by SEER organ system category, SEER classification, and related cancer SIF status within 1 year are shown in eTable 11 in Supplement 1.

## Discussion

This cohort study found that cancer SIFs reported at lung cancer screening in the NLST were significantly associated with an increased risk of being diagnosed with an extrapulmonary cancer within 1 year. The association was most evident for cancer SIFs linked to the urinary system and other cancers, including lymphoma and leukemia. Our estimates of risk associated with cancer SIFs are based on a longitudinal, cohort-based assessment of the probability of being diagnosed with cancer in a high-risk (ie, history of heavy smoking) cohort of participants undergoing lung cancer screening. To date, much of the guidance for addressing SIFs detected during lung cancer screening or other imaging has been based on expert opinion^6,7,8,9,10,11,12,13^; studies restricted to a single type of cancer^17,19^; or studies that did not adjust for potential covariates, such as cancer history.^17,18,19^

Extrapulmonary cancers are significantly associated with mortality among persons who have smoked cigarettes. Mortality from extrapulmonary cancer accounted for 22.3% of the certified deaths in the LDCT arm of the NLST.^24^ Early detection of these cancers may facilitate early treatment and potentially reduce associated morbidity and mortality. Identification of cancer SIFs associated with extrapulmonary cancers in NLST participants could be used to plan appropriate diagnostic evaluations for patients undergoing lung cancer screening.

Our analyses expand prior research.^17,18,19^ We restricted our analyses to cancer SIFs based on a comprehensive characterization of abnormalities outlined in the American College of Radiology white papers on incidental findings.^6,7,8,9,10,11,12,13^ In addition, rather than focusing on specific organs, we used broader SEER cancer categories, which yielded larger numbers of outcomes, allowing us to reliably adjust for covariates not considered in earlier analyses,^17,18,19^ including a baseline history of extrapulmonary cancer and other comorbidities. Finally, we restricted our analyses to SIFs considered clinically significant by NLST radiologists, whereas earlier publications included SIFs that were associated with minor abnormalities.^17,18,19^ These distinctions and our restriction to a 1-year time horizon from the date of SIF discovery to cancer diagnosis may explain differences between the numbers of SIFs that we report and those reported in earlier publications.^17,18,19^

Although the SIFs detected at screening may have existed prior to the first screening round, NLST entry criteria were designed to exclude participants with clinically evident disease at the time of study entry. Participants were excluded if they were treated for, or told by a physician that they had, any cancer (with the exception of certain in situ cancers) within the 5 years preceding study enrollment.^22^ More importantly, the increased risk of extrapulmonary cancer after detection of a cancer SIF was evident even after adjusting for a history of cancer diagnosis.

The Lung Imaging Reporting and Data System (Lung-RADs) was introduced in 2015 to standardize LDCT lung cancer screening reporting and was updated in 2019 and 2022.^26,27,28^ Lung-RADS incorporates the use of the S modifier to report clinically significant or potentially clinically significant (non–lung cancer) findings.^29^ However, the categories included in Lung-RADS do not contain the granularity that we incorporated into our analyses.^5^

Our findings may serve as an impetus for revising the Lung-RADS S modifier to include more specificity on classification of SIFs. Although the number of extrapulmonary cancers potentially associated with SIF detection in the NLST seem relatively small, when considered in context of the nearly 14 million people who now qualify for lung cancer screening in the US,^30^ the potential impact is large. Moreover, NLST participants were generally healthier than clinically screened patients, suggesting that the burden of extrapulmonary cancers among clinically screened patients may be higher than reported here. In addition, although the eligibility for lung cancer screening has been expanded to age 50 to 80 years and 20 pack-years of smoking,^31^ our findings may apply to current screening as well.

### Limitations

The study had several limitations. First, the number of SIFs in each category was small, and although our point estimates for digestive and breast cancer SIFs suggested an association with the linked SEER cancer categories, the 95% CLs for the risk difference measures were large and included 0. Second, 24 of 67 cancers (35.8%) diagnosed within 1 year of a cancer SIF seemed unrelated to the SEER cancer category diagnosis. The SIF may have been associated with the subsequent extrapulmonary diagnosis either because it was a general finding, such as lymphadenopathy, or it was an unrelated finding that resulted in contact with the participants’ physicians. In either case, that SIF may have led to additional workup resulting in a cancer diagnosis. Information from the NLST is not granular enough to ascertain the association. Third, SIF detection may lead to an early diagnosis of extrapulmonary cancer; however, even if findings suspicious for extrapulmonary cancers are linked with cancer diagnoses, it remains to be seen whether early detection of these extrapulmonary cancers is associated with a reduction in cancer-specific mortality. Although detection of an SIF may be beneficial to the patient, it may lead to unnecessary additional diagnostic interventions, increasing associated costs and risks. Finally, the NLST population and radiologists may not be representative of current lung cancer screening.^32^ However, we believe that the association between the cancer SIFs and cancers reported is important information for physicians currently referring patients for screening.

## Conclusions

This cohort study found a significant association between a cancer SIF on lung cancer screening in the NLST with the diagnosis of an extrapulmonary cancer in the year following the screen compared with no cancer SIF. These findings provide information on the cancer SIFs that should generate additional cancer workup when detected at lung cancer screening.

## Notes

Supplement 1. **eTable 1. **National Lung Screening Trial Inclusion and Exclusion Criteria **eTable 2. **Cancer SIFs and Related SEER Cancer Categories **eTable 3.** Information on Occupational History, Comorbidities, and History of Cancer by Cancer SIF Status **eTable 4.** Adjusted Regression Analysis Showing Marginal Risk Difference per 1000 NLST Screening Examinations Between Any Cancer for Participants With a Cancer SIF vs No Cancer SIF Over 3 Screens in the Trial **eTable 5.** Adjusted Regression Analysis Showing Marginal Risk Difference per 1000 NLST Screening Examinations Between Urinary Cancer for Participants With a Urinary Cancer SIF vs No Cancer SIF Over 3 Screens in the Trial **eTable 6.** Adjusted Regression Analysis Showing Marginal Risk Difference per 1000 NLST Screening Examinations Between Digestive Cancer for Participants With a Digestive Cancer SIF vs No Cancer SIF Over 3 Screens in the Trial **eTable 7.** Adjusted Regression Analysis Showing Marginal Risk Difference per 1000 NLST Screening Examinations Between Breast Cancer for Participants With a Breast Cancer SIF vs No Cancer SIF Over 3 Screens in the Trial **eTable 8.** Adjusted Regression Analysis Showing Marginal Risk Difference per 1000 NLST Screening Examinations Between Any Other SEER Cancer for Participants With a Nonspecific Cancer SIF vs No Cancer SIF Over 3 Screens in the Trial **eTable 9.** Detailed Information on the Type of Cancer SIF, the Occurrence of These SIFs by Screening Examination and for Each Participant, and the Number of Cancers Related to Each Cancer SIF **eTable 10.** Detailed Information on the Type of Cancer SIF, Diagnosis of a Related Extrapulmonary Cancer Within 1 Year, an Unrelated Extrapulmonary Cancer Within 1 Year of Screening Round, and Deaths Due to Extrapulmonary Cancer During the Course of the Trial **eTable 11.** The Total Number of Extrapulmonary Cancers by SEER Organ System Category, SEER Classification, and Related Cancer SIF Status Within 1 Year **eFigure.** Flowchart Describing the Study Population

Supplement 2.
Data Sharing Statement
