# Supplementary material for: Significant Incidental Findings in the National Lung Screening Trial and Diagnosis of Extrapulmonary Cancer
Source: JAMA Netw Open. 2026 Mar 31;9(3):e263398. doi: 10.1001/jamanetworkopen.2026.3398 (PMC13040399; doi:10.1001/jamanetworkopen.2026.3398)
Supplement: Supplement 1. — eTable 1. National Lung Screening Trial Inclusion and Exclusion Criteria eTable 2. Cancer SIFs and Related SEER Cancer Categories eTable 3. Information on Occupational History, Comorbidities, and History of Cancer by Cancer SIF Status eTable 4. Adjusted Regression Analysis Showing Marginal Risk Difference per 1000 NLST Screening Examinations Between Any Cancer for Participants With a Cancer SIF vs No Cancer SIF Over 3 Screens in the Trial eTable 5. Adjusted Regression Analysis Showing Marginal Risk Difference per 1000 NLST Screening Examinations Between Urinary Cancer for Participants With a Urinary Cancer SIF vs No Cancer SIF Over 3 Screens in the Trial eTable 6. Adjusted Regression Analysis Showing Marginal Risk Difference per 1000 NLST Screening Examinations Between Digestive Cancer for Participants With a Digestive Cancer SIF vs No Cancer SIF Over 3 Screens in the Trial eTable 7. Adjusted Regression Analysis Showing Marginal Risk Difference per 1000 NLST Screening Examinations Between Breast Cancer for Participants With a Breast Cancer SIF vs No Cancer SIF Over 3 Screens in the Trial eTable 8. Adjusted Regression Analysis Showing Marginal Risk Difference per 1000 NLST Screening Examinations Between Any Other SEER Cancer for Participants With a Nonspecific Cancer SIF vs No Cancer SIF Over 3 Screens in the Trial eTable 9. Detailed Information on the Type of Cancer SIF, the Occurrence of These SIFs by Screening Examination and for Each Participant, and the Number of Cancers Related to Each Cancer SIF eTable 10. Detailed Information on the Type of Cancer SIF, Diagnosis of a Related Extrapulmonary Cancer Within 1 Year, an Unrelated Extrapulmonary Cancer Within 1 Year of Screening Round, and Deaths Due to Extrapulmonary Cancer During the Course of the Trial eTable 11. The Total Number of Extrapulmonary Cancers by SEER Organ System Category, SEER Classification, and Related Cancer SIF Status Within 1 Year eFigure. Flowchart Describing the Study Population [file jamanetwopen-e263398-s001.pdf]

Supplemental Online Content

Gareen IF, Gutman R, Thangarajah M, et al. National Lung Screening Trial significant incidental findings and diagnosis of extrapulmonary cancer. *JAMA Netw Open*. 2026;9(3):263398. doi:10.1001/jamanetworkopen.2026.3398

eTable 1. National Lung Screening Trial Inclusion and Exclusion Criteria

eTable 2. Cancer SIFs and Related SEER Cancer Categories

eTable 3. Information on Occupational History, Comorbidities, and History of Cancer by Cancer SIF Status

eTable 4. Adjusted Regression Analysis Showing Marginal Risk Difference per 1000 NLST Screening Examinations Between Any Cancer for Participants With a Cancer SIF vs No Cancer-SIF Over 3 Screens in the Trial

eTable 5. Adjusted Regression Analysis Showing Marginal Risk Difference per 1000 NLST Screening Examinations Between Urinary Cancer for Participants With a Urinary Cancer-SIF vs No Cancer-SIF Over 3 Screens in the Trial

eTable 6. Adjusted Regression Analysis Showing Marginal Risk Difference per 1000 NLST Screening Examinations Between Digestive Cancer for Participants With a Digestive Cancer SIF vs No Cancer SIF Over 3 Screens in the Trial

eTable 7. Adjusted Regression Analysis Showing Marginal Risk Difference per 1000 NLST Screening Examinations Between Breast Cancer for Participants With a Breast Cancer SIF vs No Cancer SIF Over 3 Screens in the Trial

eTable 8. Adjusted Regression Analysis Showing Marginal Risk Difference per 1000 NLST Screening Examinations Between Any Other SEER Cancer for Participants With a Nonspecific Cancer SIF vs No Cancer SIF Over 3 Screens in the Trial

eTable 9. Detailed Information on the Type of Cancer SIF, the Occurrence of These SIFs by Screening Examination and for Each Participant, and the Number of Cancers Related to Each Cancer SIF

eTable 10. Detailed Information on the Type of Cancer SIF, Diagnosis of a Related Extrapulmonary Cancer Within 1 Year, an Unrelated Extrapulmonary Cancer Within 1 Year of Screening Round, and Deaths Due to Extrapulmonary Cancer During the Course of the Trial

eTable 11. The Total Number of Extrapulmonary Cancers by SEER Organ System Category, SEER Classification, and Related Cancer SIF Status Within 1 Year

eFigure. Flowchart Describing the Study Population

This supplemental material has been provided by the authors to give readers additional information about their work.

© 2026 Gareen IF et al. *JAMA Network Open*.

eTable 1. National Lung Screening Trial Inclusion and Exclusion Criteria

Inclusion:

Age 55–74 years
30 or more pack-years of cigarette smoking history (pack-years = packs per day 3 years smoked)
Former smokers: quit smoking within the previous 15 years
Ability to lie on the back with arms raised over the head
Signed informed consent form

Exclusion:

Metallic implants or devices in the chest or back, such as pacemakers or Harrington fixation rods
Treatment for, or evidence of, any cancer other than nonmelanoma skin cancer or carcinoma in situ (with the exception of transitional cell carcinoma in situ or bladder carcinoma in situ) in the 5 years prior to eligibility assessment
History of lung cancer
History of removal of any portion of the lung, excluding needle biopsy
Requirement for home oxygen supplementation
Participation in another cancer screening trial
Participation in a cancer prevention study, other than a smoking cessation study
Unexplained weight loss of more than 15 pounds in the 12 months prior to eligibility assessment
Recent hemoptysis
Pneumonia or acute respiratory infection treated with antibiotics in the 12 weeks prior to eligibility assessment
Chest CT examination in the 18 months prior to eligibility assessment

© 2026 Gareen IF et al. *JAMA Network Open*.

eTable 2. Cancer SIFs and Related SEER Cancer Categories

**Cancer-SIF**	**SEER Cancer Group**
Renal mass	Urinary System
?Renal cyst; complex renal cyst	Urinary System
Renal contour abnormality	Urinary System
Dilated renal pelvis; hydronephrosis	Urinary System
enlarged multicystic/polycystic kidneys	Urinary System
Liver lesion Not specified at <1cm Size	Digestive System
Liver: only if suspicious feature such as irregular margins, heterogeneous density. Included if description was "possible liver lesions" without enough detail to confirm benign. Include lesions for which US or MR was recommended	Digestive System
Thickening of the distal esophagus or gastric cardia	Digestive System
All focal pancreatic lesions, cystic or solid	Digestive System
Main pancreatic duct dilation, pancreatic mass	Digestive System
Esophageal mass	Digestive System
Cirrhosis	Digestive System
Suspected mass in GI tract	Digestive System
Gallbladder mass	Digestive System
Hepatomegaly	Digestive System
GB wall thickening	Digestive System
Breast – nodule, mass	Breast
Adrenal nodule or mass and not further characterized as benign	Other SEER Cancers
Thyroid nodule	Other SEER Cancers
Thymoma, thymic mass, nodule	Other SEER Cancers
Lymph node No Size, described as enlarged, adenopathy or lymphadenopathy	Other SEER Cancers
Paraspinal mass	Other SEER Cancers
Mediastinal mass (if not clearly cystic)	Other SEER Cancers
Intraabdominal lymphadenopathy	Other SEER Cancers
Splenic mass, not calcified and not clearly a cyst, requiring further follow-up	Other SEER Cancers

© 2026 Gareen IF et al. *JAMA Network Open*.

Pericardial thickening if unexplained; pericardial calcification	Other SEER Cancers
Pericardial effusion (unless specified as trace or small)	Other SEER Cancers
Lymph node >1.5 cm short axis without benign features; anything described as enlarged, adenopathy or lymphadenopathy	Other SEER Cancers
Hilum described as enlarged/large	Other SEER Cancers
Neck mass, requiring further follow-up	Other SEER Cancers
Pleural effusion	Other SEER Cancers
Suspected metastatic disease (multiple nodules)	Other SEER Cancers
Splenomegaly	Other SEER Cancers
Ascites	Other SEER Cancers
Abdominal mass or lymphadenopathy, requiring further follow-up	Other SEER Cancers
Axillary lymph nodes >= 2 cm or NOS	Other SEER Cancers
Bone lesion described as lytic/expansile	Other SEER Cancers
Chest wall mass, requiring further follow-up	Other SEER Cancers<br>Other SEER Cancers

© 2026 Gareen IF et al. *JAMA Network Open*.

eTable 3. Information on Occupational History, Comorbidities, and History of Cancer by Cancer SIF Status

Includes 26,445 participants with at least one screening examination.

Characteristic		Ever Cancer SIF		Never Cancer SIF		P-value
		Number	(%)[1]	Number	(%)	
Asbestos: Ever worked for 1 or more years?						
	Yes	84	(4.6)	1140	(4.6)	
	No	1723	(95.4)	23498	(95.4)	
Baking: Ever worked for 1 or more years?						
	Yes	35	(1.9)	558	(2.3)	
	No	1772	(98.1)	24080	(97.7)	
Butchering/meat packing: Ever worked for 1 or more years?						
	Yes	30	(1.7)	533	(2.2)	
	No	1777	(98.3)	24105	(97.8)	
Chemicals/plastics manufacturing: Ever worked for 1 or more years?						
	Yes	107	(5.9)	1526	(6.2)	
	No	1700	(94.1)	23112	(93.8)	
Coal mining: Ever worked for 1 or more years?						
	Yes	12	(0.7)	155	(0.6)	
	No	1795	(99.3)	24483	(99.4)	
Cotton/jute processing: Ever worked for 1 or more years?						
	Yes	17	(0.9)	174	(0.7)	
	No	1790	(99.1)	24464	(99.3)	
Farming: Ever worked for 1 or more years?						
	Yes	185	(10.2)	2627	(10.7)	
	No	1622	(89.8)	22011	(89.3)	
Fire fighting: Ever worked for 1 or more years?						
	Yes	29	(1.6)	446	(1.8)	
	No	1778	(98.4)	24192	(98.2)	
Flour/feed or grain milling: Ever worked for 1 or more years?						
	Yes	23	(1.3)	265	(1.1)	
	No	1784	(98.7)	24373	(98.9)	
Foundry/steel milling: Ever worked for 1 or more years?						
	Yes	84	(4.6)	1066	(4.3)	
	No	1723	(95.4)	23572	(95.7)	
Hard rock mining : Ever worked for 1 or more years?						
	Yes	14	(0.8)	187	(0.8)	
	No	1793	(99.2)	24451	(99.2)	
Painting: Ever worked for 1 or more years?						
	Yes	87	(4.8)	1274	(5.2)	
	No	1720	(95.2)	23364	(94.8)	

© 2026 Gareen IF et al. *JAMA Network Open*.

Sandblasting: Ever worked for 1 or more years?						
	Yes	33	(1.8)	420	(1.7)	
	No	1774	(98.2)	24218	(98.3)	
Welding: Ever worked for 1 or more years?						
	Yes	86	(4.8)	1409	(5.7)	
	No	1721	(95.2)	23229	(94.3)	
**Any of the above occupations?**						0.2673
	**Yes**	**484**	**(26.8)**	**6898**	**(28.0)**	
	**No**	**1323**	**(73.2)**	**17740**	**(72.0)**	
Asthma (adult): Ever diagnosed?						
	Yes	111	(6.1)	1530	(6.2)	
	No	1696	(93.9)	23108	(93.8)	
Asthma (childhood): Ever diagnosed?						
	Yes	54	(3.0)	869	(3.5)	
	No	1753	(97.0)	23769	(96.5)	
Asbestosis: Ever diagnosed?						
	Yes	18	(1.0)	254	(1.0)	
	No	1789	(99.0)	24384	(99.0)	
Bronchiectasis: Ever diagnosed?						
	Yes	62	(3.4)	786	(3.2)	
	No	1745	(96.6)	23852	(96.8)	
Chronic bronchitis: Ever diagnosed?						
	Yes	184	(10.2)	2380	(9.7)	
	No	1623	(89.8)	22258	(90.3)	
COPD: Ever diagnosed?						
	Yes	92	(5.1)	1244	(5.0)	
	No	1715	(94.9)	23394	(95.0)	
Diabetes: Ever diagnosed?						
	Yes	179	(9.9)	2385	(9.7)	
	No	1628	(90.1)	22253	(90.3)	
Emphysema: Ever diagnosed?						
	Yes	161	(8.9)	1872	(7.6)	
	No	1646	(91.1)	22766	(92.4)	
Fibrosis of the lung: Ever diagnosed?						
	Yes	4	(0.2)	65	(0.3)	
	No	1803	(99.8)	24573	(99.7)	
Heart disease or heart attack: Ever diagnosed?						
	Yes	229	(12.7)	3189	(12.9)	
	No	1578	(87.3)	21449	(87.1)	
Hypertension: Ever diagnosed?						
	Yes	724	(40.1)	8570	(34.8)	
	No	1083	(59.9)	16068	(65.2)	
Pneumonia: Ever diagnosed?						
	Yes	395	(21.9)	5493	(22.3)	

© 2026 Gareen IF et al. *JAMA Network Open*.

	No	1412	(78.1)	19145	(77.7)	
Sarcoidosis: Ever diagnosed?						
	Yes	4	(0.2)	42	(0.2)	
	No	1803	(99.8)	24596	(99.8)	
Silicosis: Ever diagnosed?						
	Yes	2	(0.1)	27	(0.1)	
	No	1805	(99.9)	24611	(99.9)	
Stroke: Ever diagnosed?						
	Yes	55	(3.0)	695	(2.8)	
	No	1752	(97.0)	23943	(97.2)	
Tuberculosis: Ever diagnosed?						
	Yes	20	(1.1)	257	(1.0)	
	No	1787	(98.9)	24381	(99.0)	
**Diagnosed with any of the above diseases?**						**0.0122**
	**Yes**	**1239**	**(68.6)**	**16180**	**(65.7)**	
	**No**	**568**	**(31.4)**	**8458**	**(34.3)**	
Bladder Cancer: Ever diagnosed?						
	Yes	8	(0.4)	104	(0.4)	
	No	1799	(99.6)	24534	(99.6)	
Breast Cancer: Ever diagnosed?						
	Yes	32	(1.8)	317	(1.3)	
	No	1775	(98.2)	24321	(98.7)	
Cervical Cancer: Ever diagnosed?						
	Yes	21	(1.2)	339	(1.4)	
	No	1786	(98.8)	24299	(98.6)	
Colorectal Cancer: Ever diagnosed?						
	Yes	8	(0.4)	100	(0.4)	
	No	1799	(99.6)	24538	(99.6)	
Esophageal Cancer: Ever diagnosed?						
	Yes	1	(0.1)	11	(<0.01)	
	No	1806	(99.9)	24627	(99.9)	
Kidney Cancer: Ever diagnosed?						
	Yes	8	(0.4)	29	(0.1)	
	No	1799	(99.6)	24609	(99.9)	
Larynx Cancer: Ever diagnosed?						
	Yes	0	(0.0)	20	(0.1)	
	No	1807	(100.0)	24618	(99.9)	
Nasal Cancer: Ever diagnosed?						
	Yes	1	(0.1)	10	(<0.01)	
	No	1806	(99.9)	24628	(99.9)	
Oral Cancer: Ever diagnosed?						
	Yes	3	(0.2)	47	(0.2)	
	No	1804	(99.8)	24591	(99.8)	
Pancreatic Cancer: Ever diagnosed?						

© 2026 Gareen IF et al. *JAMA Network Open*.

	Yes	0	(0.0)	4	(<0.01)	
	No	1807	(100.0)	24634	(99.9)	
Pharynx Cancer: Ever diagnosed?						
	Yes	0	(0.0)	3	(<0.01)	
	No	1807	(100.0)	24635	(99.9)	
Stomach Cancer: Ever diagnosed?						
	Yes	2	(0.1)	10	(<0.01)	
	No	1805	(99.9)	24628	(99.9)	
Thyroid Cancer: Ever diagnosed?						
	Yes	4	(0.2)	31	(0.1)	
	No	1803	(99.8)	24607	(99.9)	
Transitional Cell Cancer: Ever diagnosed?						
	Yes	1	(0.1)	12	(<0.01)	
	No	1806	(99.9)	24626	(99.9)	
**Diagnosed with any of the above cancers?**						0.1840
	**Yes**	**84**	**(4.6)**	**988**	**(4.0)**	
	**No**	**1723**	**(95.4)**	**23650**	**(96.0)**	
**Total**		1807	**(100.0)**	24638	**(100.0)**	

© 2026 Gareen IF et al. *JAMA Network Open*.

eTable 4. Adjusted Regression Analysis Showing Marginal Risk Difference per 1000 NLST Screening Examinations Between Any Cancer for Participants With a Cancer SIF vs No Cancer SIF Over 3 Screens in the Trial

Includes 75,104 screening examinations. At least one cancer-SIF was detected at 2265 screening examinations, and 1025 participants with at least 1 extrapulmonary cancer diagnosed are included. Detailed Regression Results. This table shows the marginal average risk difference for all variables individually.

	Estimate	Lower 95%	Upper 95%	P-value
Age (+1)	0.00082	0.00064	0.00100	<0.001
Any Previous cancer (Yes vs No)	0.00375	-0.00108	0.00858	0.128
Any cancer sif (Yes vs No)	0.01389	0.00703	0.02075	<0.001
Smoking Status (Current vs Former)	0.00176	0.00004	0.00347	0.045
Any previous diagnosis? (Yes vs No)	0.00140	-0.00037	0.00317	0.122
Education Status (11th grade or less vs Associates degree/some college)	-0.00211	-0.00568	0.00145	0.245
Education Status (High school graduate/GED vs Associates degree/some college)	-0.00077	-0.00322	0.00168	0.538
Education Status (Post high school training, excluding college vs Associates degree/some college)	-0.00211	-0.00485	0.00062	0.13
Education Status (Bachelor’s Degree vs Associates degree/some college)	0.00279	-0.00006	0.00564	0.055
Education Status (Graduate School vs Associates degree/some college)	0.00053	-0.00234	0.00340	0.718
Education Status (Other vs Associates degree/some college)	-0.00013	-0.00624	0.00597	0.966
Ethnicity (Not Hispanic or Latino vs Hispanic/Latino)	0.00004	-0.00656	0.00663	0.992
Ethnicity (Other vs Hispanic/Latino)	0.00456	-0.01263	0.02176	0.603
Gender (Females vs Males)	-0.00401	-0.00585	-0.00217	<0.001
Martial status (Never married vs Married or living as married)	0.00272	-0.00177	0.00722	0.235
Martial status (Windowed vs Married or living as married)	-0.00073	-0.00394	0.00249	0.658
Martial status (Separated vs Married or living as married)	0.00203	-0.00631	0.01036	0.634
Martial status (Divorced vs Married or living as married)	-0.00067	-0.00291	0.00157	0.559
Martial status (Other vs Married or living as married)	-0.00401	-0.01765	0.00963	0.564
Pack-Years (+1)	-0.00002	-0.00006	0.00001	0.223
Race (Black vs White)	0.00045	-0.00395	0.00485	0.842
Race (Other vs White)	-0.00430	-0.00778	-0.00082	0.015
Time (1 vs 0)	-0.00032	-0.00232	0.00169	0.757
Time (2 vs 0)	-0.00021	-0.00222	0.00180	0.837
Time (1 vs 0) where any cancer sif = 0	0.00317	-0.01389	0.02024	0.716
Time (1 vs 0) where any cancer sif = 1	-0.01067	-0.02699	0.00565	0.2
Time (2 vs 0) where any cancer sif = 1	0.00031	-0.00174	0.00236	0.766
Time (2 vs 0) where any cancer sif = 0	0.00065	-0.00140	0.00270	0.532
Any work exposure? (Yes vs No)	0.00036	-0.00162	0.00234	0.719

© 2026 Gareen IF et al. *JAMA Network Open*.

eTable 5. Adjusted Regression Analysis Showing Marginal Risk Difference per 1000 NLST Screening Examinations Between Urinary Cancer for Participants With a Urinary Cancer-SIF vs No Cancer-SIF Over 3 Screens in the Trial

Includes 75,104 screening examinations. At least one cancer-SIF was detected at 2265 screening examinations, and 1025 participants with at least 1 extrapulmonary cancer diagnosed are included. Detailed Regression Results. This table shows the marginal average risk difference for all variables individually.

	Estimate	Lower 95%	Upper 95%	P-value
Age (+1)	0.00019	0.00012	0.00026	<0.001
Any previous cancer? (Yes vs No)	0.00118	-0.00090	0.00326	0.268
Smoking Status (Current vs Former)	0.00067	0.00000	0.00133	0.049
Any previous diagnosis? (Yes vs No)	0.00017	-0.00051	0.00085	0.626
Education Status (11th grade or less vs Associates degree/some college)	-0.00069	-0.00184	0.00046	0.238
Education Status (High school graduate/GED vs Associates degree/some college)	0.00012	-0.00081	0.00105	0.799
Education Status (Post high school training, excluding college vs Associates degree/some college)	0.00061	-0.00056	0.00178	0.308
Education Status (Bachelor’s Degree vs Associates degree/some college)	0.00025	-0.00079	0.00129	0.638
Education Status (Graduate School vs Associates degree/some college)	-0.00021	-0.00122	0.00080	0.684
Education Status (Other vs Associates degree/some college)	0.00190	-0.00118	0.00499	0.226
Sex (Females vs Males)	-0.00105	-0.00171	-0.00039	0.002
Pack-Years (+1)	0.00001	-0.00001	0.00002	0.277
Race (Black vs White)	-0.00063	-0.00206	0.00081	0.391
Race (Other vs White)	-0.00110	-0.00222	0.00002	0.055
Time (1 vs 0)	-0.00020	-0.00097	0.00057	0.608
Time (2 vs 0)	-0.00024	-0.00102	0.00054	0.548
Any Urinary SIF (Yes vs No)	0.01703	0.00855	0.02550	<0.001
Any work exposure? (Yes vs No)	0.00005	-0.00068	0.00077	0.903

© 2026 Gareen IF et al. *JAMA Network Open*.

eTable 6. Adjusted Regression Analysis Showing Marginal Risk Difference per 1000 NLST Screening Examinations Between Digestive Cancer for Participants With a Digestive Cancer SIF vs No Cancer SIF Over 3 Screens in the Trial

Includes 75,104 screening examinations. At least one cancer-SIF was detected at 2265 screening examinations, and 1025 participants with at least 1 extrapulmonary cancer diagnosed are included. Detailed Regression Results. This table shows the marginal average risk difference for all variables individually.

	Estimate	Lower 95%	Upper 95%	P-value
Any Digestive SIF (Yes vs No)	0.00502	-0.00133	0.01136	0.121
Age (+1)	0.00012	0.00004	0.00019	0.002
Any previous cancer? (Yes vs No)	0.00107	-0.00113	0.00327	0.342
Smoking Status (Current vs Former)	0.00021	-0.00049	0.00090	0.560
Any previous diagnosis? (Yes vs No)	0.00011	-0.00062	0.00084	0.767
Education Status (11th grade or less vs Associates degree/some college)	-0.00030	-0.00190	0.00129	0.710
Education Status (High school graduate/GED vs Associates degree/some college)	-0.00076	-0.00177	0.00025	0.139
Education Status (Post high school training,excluding college vs Associates degree/some college)	-0.00008	-0.00133	0.00116	0.897
Education Status (Bachelor’s Degree vs Associates degree/some college)	-0.00071	-0.00179	0.00036	0.194
Education Status (Graduate School vs Associates degree/some college)	-0.00037	-0.00155	0.00080	0.536
Education Status (Other vs Associates degree/some college)	0.00137	-0.00191	0.00465	0.412
Sex (Females vs Males)	-0.00104	-0.00175	-0.00032	0.004
Pack-Years (+1)	-0.00001	-0.00002	0.00001	0.279
Race (Black vs White)	0.00032	-0.00148	0.00212	0.729
Race (Other vs White)	0.00072	-0.00113	0.00256	0.446
Time (1 vs 0)	-0.00027	-0.00111	0.00057	0.527
Time (2 vs 0)	-0.00041	-0.00124	0.00042	0.327
Any work exposure? (Yes vs No)	0.00000	-0.00079	0.00079	0.993

© 2026 Gareen IF et al. *JAMA Network Open*.

eTable 7. Adjusted Regression Analysis Showing Marginal Risk Difference per 1000 NLST Screening Examinations Between Breast Cancer for Participants With a Breast Cancer SIF vs No Cancer SIF Over 3 Screens in the Trial

Includes 75,104 screening examinations. At least one cancer-SIF was detected at 2265 screening examinations, and 1025 participants with at least 1 extrapulmonary cancer diagnosed are included. Detailed Regression Results. This table shows the marginal average risk difference for all variables individually.

	Estimate	Lower 95%	Upper 95%	P-value
Age (+1)	0.00000	-0.00006	0.00007	0.943
Any Breast SIF (Yes vs No)	0.01230	-0.00387	0.02848	0.136
Any previous cancer? (Yes vs No)	0.00259	0.00028	0.00490	0.028
Smoking Status (Current vs Former)	-0.00015	-0.00080	0.00050	0.645
Any previous diagnosis? (Yes vs No)	0.00063	-0.00001	0.00128	0.055
Education Status (11th grade or less vs Associates degree/some college)	0.00066	-0.00127	0.00258	0.502
Education Status (High school graduate/GED vs Associates degree/some college)	0.00018	-0.00081	0.00117	0.726
Education Status (Post high school training, excluding college vs Associates degree/some college)	-0.00064	-0.00164	0.00036	0.212
Education Status (Bachelor's Degree vs Associates degree/some college)	0.00033	-0.00072	0.00139	0.535
Education Status (Graduate School vs Associates degree/some college)	-0.00035	-0.00134	0.00064	0.491
Education Status (Other vs Associates degree/some college)	-0.00149	-0.00281	-0.00017	0.027
Pack-Years (+1)	-0.00003	-0.00005	-0.00001	0.002
Race (Black vs White)	-0.00056	-0.00192	0.00079	0.413
Race (Other vs White)	0.00010	-0.00152	0.00172	0.904
Time (1 vs 0)	-0.00006	-0.00084	0.00071	0.871
Time (2 vs 0)	-0.00001	-0.00080	0.00078	0.99
Any work exposure? (Yes vs No)	-0.00174	-0.00231	-0.00117	<0.001

© 2026 Gareen IF et al. *JAMA Network Open*.

eTable 8. Adjusted Regression Analysis Showing Marginal Risk Difference per 1000 NLST Screening Examinations Between Any Other SEER Cancer for Participants With a Nonspecific Cancer SIF vs No Cancer SIF Over 3 Screens in the Trial

Includes 75,104 screening examinations. At least one cancer-SIF was detected at 2265 screening examinations, and 1025 participants with at least 1 extrapulmonary cancer diagnosed are included. Detailed Regression Results. This table shows the marginal average risk difference for all variables individually.

	Estimate	Lower 95%	Upper 95%	P-value
Age (+1)	0.00052	0.00039	0.00066	<0.001
Any previous cancer? (Yes vs No)	0.00027	-0.00339	0.00393	0.885
Smoking Status (Current vs Former)	0.00138	0.00008	0.00267	0.037
Any previous diagnosis? (Yes vs No)	0.00054	-0.00079	0.00188	0.427
Education Status (11th grade or less vs Associates degree/some college)	-0.00149	-0.00405	0.00107	0.255
Education Status (High school graduate/GED vs Associates degree/some college)	-0.00002	-0.00190	0.00186	0.983
Education Status (Post high school training, excluding college vs Associates degree/some college)	-0.00174	-0.00373	0.00026	0.088
Education Status (Bachelor's Degree vs Associates degree/some college)	0.00207	-0.00007	0.00420	0.057
Education Status (Graduate School vs Associates degree/some college)	0.00090	-0.00126	0.00307	0.414
Education Status (Other vs Associates degree/some college)	-0.00200	-0.00608	0.00208	0.337
Ethnicity (Not Hispanic or Latino vs Hispanic/Latino)	-0.00240	-0.00812	0.00332	0.411
Ethnicity (Other vs Hispanic/Latino)	0.00506	-0.01071	0.02083	0.529
Sex (Females vs Males)	-0.00676	-0.00803	-0.00549	<0.001
Martial status (Never married vs Married or living as married)	0.00152	-0.00194	0.00499	0.389
Martial status (Windowed vs Married or living as married)	-0.00194	-0.00433	0.00046	0.113
Martial status (Separated vs Married or living as married)	0.00176	-0.00461	0.00813	0.588
Martial status (Divorced vs Married or living as married)	-0.00103	-0.00273	0.00066	0.232
Martial status (Other vs Married or living as married)	-0.00296	-0.01271	0.00679	0.552
Any non-specific cancer sif (Yes vs No)	0.01383	0.00346	0.02421	0.009
Pack-Years (+1)	-0.00002	-0.00004	0.00001	0.265
Race (Black vs White)	0.00158	-0.00211	0.00526	0.402
Race (Other vs White)	-0.00457	-0.00656	-0.00257	<0.001
Time (1 vs 0)	0.00033	-0.00116	0.00182	0.664
Time (2 vs 0)	0.00109	-0.00046	0.00263	0.167
Any work exposure? (Yes vs No)	0.00045	-0.00099	0.00188	0.541

© 2026 Gareen IF et al. *JAMA Network Open*.

eTable 9. Detailed Information on the Type of Cancer SIF, the Occurrence of These SIFs by Screening Examination and for Each Participant, and the Number of Cancers Related to Each Cancer SIF

	N SIFs across all 3 screening examinations	N people with that SIF across 3 screening examinations	N Related cancers per supp table 1 within 1 year SEER_Class	N Related cancers per supp table 1 within 1 year SEER_Cat
Urinary Cancer related SIFs[1]<br>(N=655 unique participants with urinary SIFs – 164 participants had urinary SIFs at different timepoints)<br>Note: 33 participants had two urinary SIFs at the same timepoint and one participant with 3 urinary SIFs at the same timepoint.				
Renal mass	647	524	18 (Kidney and Renal Pelvis)	18
?Renal cyst; complex renal cyst	134	117	1 (Ureter)	1
Renal contour abnormality	39	39	1 (Kidney and Renal Pelvis)	1
Dilated renal pelvis; hydronephrosis	30	27	-	-
enlarged multicystic/polycystic kidneys	3	3	-	-
Digestive Cancer related SIFs[2]<br>(N=537 unique participants with Digestive SIFs – 109 participants had digestive SIFs at different timepoints)<br>Note: 58 participants had two digestive SIFs at the same timepoint and two participant with 3 digestive SIFs at the same timepoint.				
Liver lesion Not specified at <1cm Size	420	352	3 (Sigmoid Colon)<br>1 (Large Intestine, NOS)	4
Liver: only if suspicious feature such as irregular margins, heterogeneous density. Included if description was "possible liver lesions" without enough detail to confirm benign. Include lesions for which US or MR was recommended	143	136	1 (Sigmoid Colon)	1
Thickening of the distal esophagus or gastric cardia	45	42	-	-
All focal pancreatic lesions, cystic or solid	33	27	-	-
Main pancreatic duct dilation, pancreatic mass	17	16	1 (Pancreas)	1
Esophageal mass	10	9	-	-
Cirrhosis	22	11	-	-
Suspected mass in GI tract	8	7	-	-
Gallbladder mass	6	4	-	-
Hepatomegaly	3	3	-	-
GB wall thickening	1	1	-	-
Breast Cancer related SIFs<br>(N=121 unique participants with Breast SIFs – 40 participants had breast SIFs at different timepoints)				
Breast – nodule, mass	161	121	3 (Breast)	3
Other SEER Cancers[3]<br>(N=652 unique participants with non-specific SIFs – 129 participants had non-specific SIFs reported at different timepoints)<br>Note: 44 participants had two non-specific SIFs at the same timepoint, 4 with three non-specific SIFs and one participants with four non-specific SIFs at the same timepoint.				
Adrenal nodule or mass and not further characterized as benign	265	220	-	-
Thyroid nodule	92	79	2 (Thyroid)	2 (Endocrine System)
Thymoma, thymic mass, nodule	12	12	1 (Other Endocrine including Thymus)	1 (Endocrine System)
Lymph node No Size, described as enlarged, adenopathy or lymphadenopathy	110	103	1 (Prostate)	1 (Male Genital System)
			1 (Hodgkin – Nodal)<br>3 (NHL – Nodal)<br>1 (NHL – Extranodal)	5 (Lymphoma)
Paraspinal mass	19	13	1 (Oropharynx)	1 (Oral Cavity and Pharynx)
Mediastinal mass (if not clearly cystic)	51	43	-	-
Intraabdominal lymphadenopathy	4	4	-	-
Splenic mass, not calcified and not clearly a cyst, requiring further follow-up	1	1	-	-
Pericardial thickening if unexplained; pericardial calcification	60	53	1 (Prostate)	1 (Male Genital System)
Pericardial effusion (unless specified as trace or small)	69	50	1 (Misc)	1 (Miscellaneous)
			1 (Prostate)	1 (Male Genital System)
Lymph node >1.5 cm short axis without benign features; anything described as enlarged, adenopathy or lymphadenopathy	6	6	-	-
Hilum described as enlarged/large	4	4	-	-
Neck mass, requiring further follow-up	1	1	-	-

© 2026 Gareen IF et al. *JAMA Network Open*.

Pleural effusion	21	21	-	-
Suspected metastatic disease (multiple nodules)	16	15	1 (Miscellaneous)	1 (Miscellaneous)
Splenomegaly	18	15	-	-
Ascites	16	14	-	-
Abdominal mass or lymphadenopathy, requiring further follow-up	13	13	-	-
Axillary lymph nodes >= 2 cm or NOS	52	44	2 (NHL – Nodal)<br>2 (NHL – Extranodal)	4 (Lymphoma)
Bone lesion described as lytic/expansile	5	3	-	-
Chest wall mass, requiring further follow-up	1	1	-	-

[1]One participant had two related SIFs and was diagnosed with urinary cancer within 1 year of screening.

[2]One participant had two related SIFs and was diagnosed with a digestive cancer within 1 year of screening.

[3]Two participants had 2 SIFs and were diagnosed with a cancer within 1 year of screening.

© 2026 Gareen IF et al. *JAMA Network Open*.

eTable 10. Detailed Information on the Type of Cancer SIF, Diagnosis of a Related Extrapulmonary Cancer Within 1 Year, an Unrelated Extrapulmonary Cancer Within 1 Year of Screening Round, and Deaths Due to Extrapulmonary Cancer During the Course of the Trial

	Urinary Cancer within 1 yr		Other extrapulmonary cancer within 1 yr		No cancer within 1 yr	
	Alive	Extrapulmonary Cancer death	Alive	Extrapulmonary Cancer death	Alive	Extrapulmonary Cancer death
Renal mass	17	1	5	3	444	10
?Renal cyst; complex renal cyst	1	-		1	106	-
Renal contour abnormality	-	1	1		37	-
Dilated renal pelvis; hydronephrosis	-	-	-	-	22	1
enlarged multicystic/polycystic kidneys	-	-	-	-	3	-
	Digestive Cancer within 1 yr		Other extrapulmonary cancer within 1 yr		No cancer within 1 yr	
	Alive	Extrapulmonary Cancer death	Alive	Extrapulmonary Cancer death	Alive	Extrapulmonary Cancer death
Liver lesion Not specified at <1cm Size	2	2	2	1	320	8
Liver: only if suspicious feature such as irregular margins, heterogeneous density. Included if description was “possible liver lesions” without enough detail to confirm benign. Include lesions for which US or MR was recommended	-	1	2	1	117	8
Thickening of the distal esophagus or gastric cardia	-	-	-	-	35	3
All focal pancreatic lesions, cystic or solid	-	-	-	-	23	1
Main pancreatic duct dilation, pancreatic mass	-	1	-	-	15	-
Esophageal mass	-	-	-	-	7	2
Cirrhosis	-	-	-	-	10	-
Suspected mass in GI tract	-	-	1	-	6	-
Gallbladder mass	-	-	-	-	3	1
Hepatomegaly	-	-	-	-	2	-
GB wall thickening	-	-	-	-	1	-
	Breast Cancer within 1 yr		Other extrapulmonary cancer within 1 yr		No cancer within 1 yr	
	Alive	Extrapulmonary Cancer death	Alive	Extrapulmonary Cancer death	Alive	Extrapulmonary Cancer death
Breast – nodule, mass	2	1	1	1	111	2
	Non-Urinary, GI, Or Breast, Cancer within 1 yr		Other extrapulmonary cancer within 1 yr		No cancer within 1 yr	
	Alive	Extrapulmonary Cancer death	Alive	Extrapulmonary Cancer death	Alive	Extrapulmonary Cancer death
Adrenal nodule or mass and not further characterized as benign	-	-	1	1	194	8
Thyroid nodule[1]	2	-	-	1	70	3
Thymoma, thymic mass, nodule[1]	1	-	-	-	11	-
Lymph node No Size, described as enlarged, adenopathy or lymphadenopathy[2]	5 (Lymphoma)	-	-	-	86	2
Paraspinal mass[3]	1 (Oral Cavity and Pharynx)	-	1	-	10	-
Mediastinal mass (if not clearly cystic)	-	-	-	-	37	3
Intraabdominal lymphadenopathy	-	-	-	-	3	-
Splenic mass, not calcified and not clearly a cyst, requiring further follow-up	-	-	-	-	1	-
Pericardial thickening if unexplained; pericardial calcification[4]	-	1	-	-	46	2

© 2026 Gareen IF et al. *JAMA Network Open*.

Pericardial effusion (unless specified as trace or small)[45]	-	2 (One participant diagnosed with Miscellaneous & another participant with Male Genital System cancer)	-	-	44	1
Lymph node >1.5 cm short axis without benign features; anything described as enlarged, adenopathy or lymphadenopathy	-	-	-	-	6	-
Hilum described as enlarged/large	-	-	-	-	4	-
Neck mass, requiring further follow-up	-	-	-	-	1	-
Pleural effusion	-	-	-	-	14	1
Suspected metastatic disease (multiple nodules)[6]	-	1 (Miscellaneous)	-	-	14	-
Splenomegaly	-	-	-	1	12	1
Ascites	-	-	-	-	6	2
Abdominal mass or lymphadenopathy, requiring further follow-up	-	-	-	-	13	-
Axillary lymph nodes >= 2 cm or NOS[7]	4 (Lymphoma)	-	1	-	37	1
Bone lesion described as lytic/expansile	-	-	1	-	1	1
Chest wall mass, requiring further follow-up	-	-	-	-	1	-

[1]Participants diagnosed with endocrine cancers and were alive by the end of the study.

[2]Five participants diagnosed with Lymphoma cancers and were alive. One participant was diagnosed with a prostate cancer and died with another cause of death (I251).

[3]One participant was diagnosed with an oral cavity and pharynx cancer and was alive by the end of the study.

[4]One participant was diagnosed with prostate cancer and passed away due to neoplasms of the central nervous (C61).

[5]One participant was diagnosed with an miscellaneous cancer and passed away due to neoplasm of digestive organs (C229).

[6]One participant was diagnosed with an miscellaneous cancer and passed away due to cancer without specification of site (C80).

[7]Participants diagnosed with lymphoma cancers and were alive by the end of the study.

© 2026 Gareen IF et al. *JAMA Network Open*.

eTable 11. The Total Number of Extrapulmonary Cancers by SEER Organ System Category, SEER Classification, and Related Cancer SIF Status Within 1 Year

SEER Organ System Category	N	SEER Classification	Number of participants with SEER classification	Number of participants with no cancer-related SIF within 1 year	Number of participants with cancer-related SIF within 1 year[1]
Urinary Cancer	148	Urinary Bladder	90	90	
		Kidney and Renal Pelvis	51	33	18
		Ureter	7	6	1
Digestive Cancer	169	Esophagus	13	13	
		Stomach	10	10	
		Small Intestine	3	3	
		Cecum	9	9	
		Appendix	1	1	
		Ascending Colon	5	5	
		Hepatic Flexure	5	5	
		Transverse Colon	6	6	
		Descending Colon	6	6	
		Sigmoid Colon	28	25	3
		Large Intestine, NOS	4	3	1
		Rectosigmoid Junction	4	4	
		Rectum	14	14	
		Anus, Anal Canal and Anorectum	5	5	
		Liver	8	8	
		Other Biliary	3	3	
		Pancreas	38	37	1
		Retroperitoneum	1	1	
		Peritoneum, Omentum and Mesentery	4	4	
		Other Digestive Organs	2	2	
Breast	152	Breast	152	149	3
Other Cancers	*579*			*563*	*16*
Myeloma	12	Myeloma	12	12	
Male Genital System	311	Prostate	306	304	2
		Testis	3	3	
		Penis	2	2	
Oral Cavity and Pharynx	38	Tongue	13	13	
		Salivary Gland	2	2	
		Floor of Mouth	7	7	
		Gum and Other Mouth	4	4	
		Nasopharynx	1	1	
		Tonsil	4	4	
		Oropharynx	2	1	1
		Hypopharynx	2	2	
		Other OralCavity and Pharynx	3	3	
Female Genital System	29	Cervix Uteri	3	3	
		Corpus Uteri	12	12	
		Uterus, NOS	1	1	
		Ovary	12	12	
		Vulva	1	1	
Leukemia	25	Chronic Lymphocytic Leukemia	8	8	
		Other Lymphocytic Leukemia	3	3	
		Acute Myeloid Leukemia	9	9	
		Chronic Myeloid Leukemia	2	2	
		Acute Monocytic Leukemia	1	1	
		Other Acute Leukemia	1	1	
		Aleukemic, subleukemic and NOS	1	1	
Brain and other nervous systems	17	Brain	15	15	

© 2026 Gareen IF et al. *JAMA Network Open*.

		Cranial Nerves Other Nervous System	2	2	
Skin excluding Basal and Squamous	48	Melanoma of the Skin	43	43	
		Other Non-Epithelial Skin	5	5	
Soft Tissue including Heart	4	Soft Tissue including Heart	4	4	
Mesothelioma	1	Mesothelioma	1	1	
Endocrine System	25	Thyroid	23	21	2
		Other Endocrine including Thymus	2	1	1
Lymphoma	47	Hodgkin - Nodal	3	2	1
		NHL - Nodal	26	21	5
		NHL - Extranodal	18	16	2
Eye and Orbit	1	Eye and Orbit	1	1	
Bones and Joints	1	Bones and Joints	1	1	
Miscellaneous	20	Miscellaneous	20	18	2

[1]24 participants have SIFs associated with unrelated cancers.

© 2026 Gareen IF et al. *JAMA Network Open*.

eFigure. Flowchart Describing the Study Population

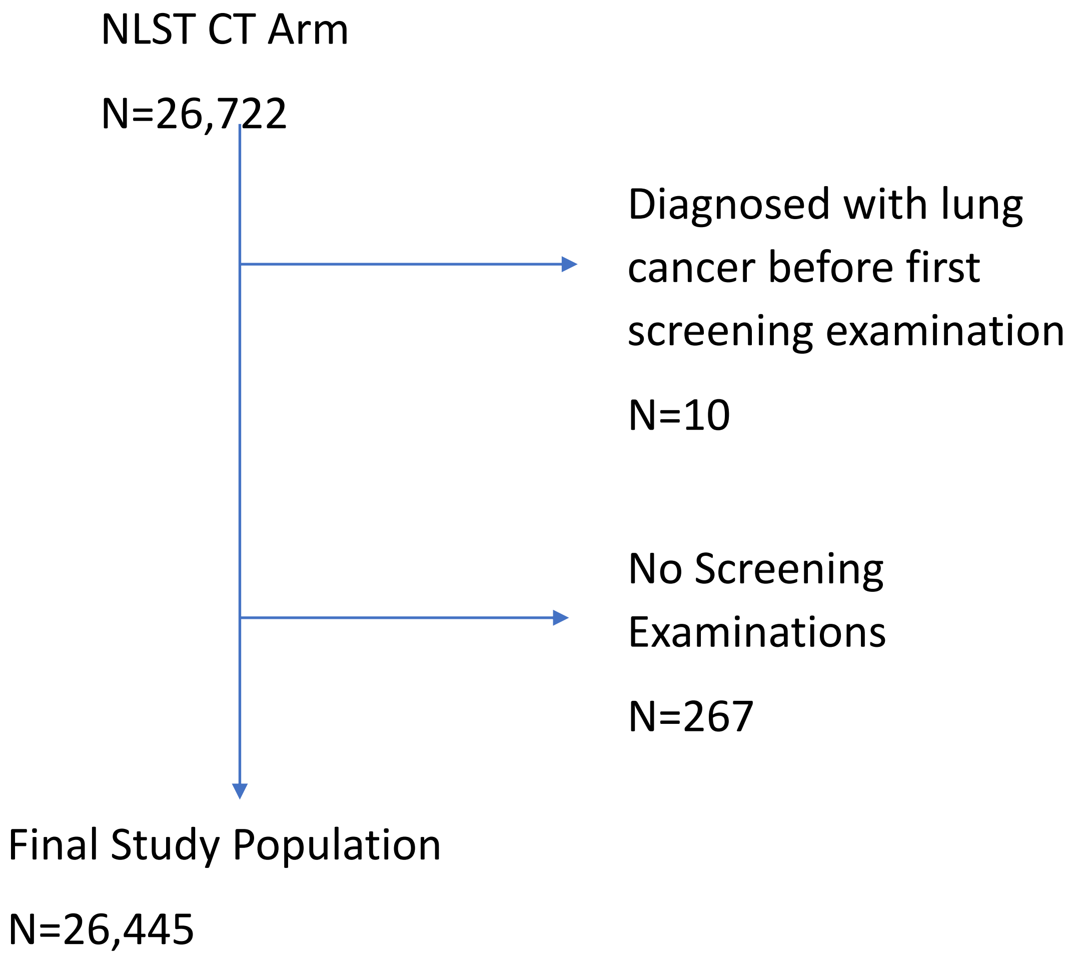

© 2026 Gareen IF et al. *JAMA Network Open*.
