# Supplementary material for: Significant Incidental Findings in the National Lung Screening Trial and Diagnosis of Extrapulmonary Cancer
Source: JAMA Netw Open. 2026 Mar 31;9(3):e263398. doi: 10.1001/jamanetworkopen.2026.3398 (PMC13040399; doi:10.1001/jamanetworkopen.2026.3398)
Supplement: Supplement 2. — Data Sharing Statement [file jamanetwopen-e263398-s002.pdf]

Data Sharing Statement

Data
Data available: No

Additional Information
Explanation for why data not available: National Lung Screening Trial data are available through a data request to https://cdas.cancer.gov/nlst/
